# Secondary Renal Injury and Encephalopathy Syndrome Caused by Systemic Lupus Erythematosus With Overlapping Sjögren's Syndrome in Children: A Case Report

**DOI:** 10.1155/carm/6921533

**Published:** 2025-07-30

**Authors:** Xiao-Ling Li, Chun-Lei Liu, Yan Ma

**Affiliations:** ^1^Department of Pediatrics, Children's Hospital Affiliated to Shandong University (Jinan Children's Hospital), No. 23976 Jingshi Road, Huaiyin District, Jinan 250000, China; ^2^Department of Information Technology, Children's Hospital Affiliated to Shandong University (Jinan Children's Hospital), No. 23976 Jingshi Road, Huaiyin District, Jinan 250000, China; ^3^Department of Pediatrics, Provincial Third Hospital, No. 11 Wuyingshan Road, Tianqiao District, Jinan 250000, China

**Keywords:** children, encephalopathy, renal injury, Sjögren's syndrome, systemic lupus erythematosus

## Abstract

Systemic lupus erythematosus (SLE) and Sjögren's syndrome (SS) are chronic, multisystem disorders. When the two coexist, the manifestations become more complex and diverse, and early diagnosis and treatment are a key to improving the patient prognosis. However, to date, only scarce reports have been published, especially overlap syndrome. We review the diagnosis and treatment process of a case of acute renal injury and encephalopathy syndrome secondary to SLE with overlapping SS to gain a comprehensive understanding of the disease with review of the current literature.

## 1. Introduction

Although systemic lupus erythematosus (SLE) and Sjögren's syndrome (SS) are two different autoimmune diseases, the course of them exhibits a correlation, sharing a common immunogenetic background. SLE is a chronic systemic autoimmune disorder characterized by the presence of multiple autoantibodies in the serum, notably antinuclear antibodies (ANAs). Its typical manifestations encompass kidneys, cardiovascular system, nerves, skeletal muscles, and skin system [1]. SS, on the other hand, is a chronic inflammatory autoimmune disease primarily affecting exocrine glands such as the lacrimal and salivary glands. The most prevalent symptoms are dry mouth and eyes, with potential involvement of any system or organ. It can exist independently or be combined with other connective tissue diseases, such as SLE and rheumatoid arthritis [2]. As early as 1959, researchers first found that SLE coexist with SS, and the incidence rate was about 9%–31% [3, 4]. However, the presentations become even more intricate and varied, so it is difficult to distinguish the primary disease when the two are concurrent. Here, we present a case of acute renal injury and encephalopathy syndrome secondary to SLE with overlapping SS (SLE-SS) to gain a comprehensive understanding of the association of diseases and the choice of treatment schemes after the occurrence of complications with review of the current literature.

## 2. Case Presentation

### 2.1. Symptom Onset

A 13-year-old girl presented with swelling of both eyes for one month. The child experienced swelling in both eyes, accompanied by blurred vision, without photosensitivity, morning stiffness, or other discomforts 1 month ago. Examination at a local hospital showed ANAs > 500.0 AU/mL, complement C3 0.47 g/L, complement C4 0.02 g/L, urine total protein 3906 mg/L, urine microalbumin 2394 mg/L, and urine protein/creatinine 2639 mg/g. Due to concern of lupus nephritis, the patient was transferred to tertiary care hospital for further management. Her past medical history was unremarkable. The birth history and feeding history were uneventful. There was no history of similar illness in the family.

### 2.2. Physical Examination

The child suffered chemosis, blurred vision, dry mouth, low breathing sound, abdominal swelling, and both lower extremities, with no abnormalities found on neurological examination.

### 2.3. Auxiliary Examination

Laboratory examination showed massive proteinuria, including urinary protein 3+, urinary microalbumin creatinine ratio (ACR) (88.54 mg/mmol, normal range: 0–2.5 mg/mmol), 24 h urinary protein (727.55 mg/24 h, normal range: 28–141 mg/24 h), hyperlipidemia (7.73 mmol/L, normal range: 3.1–5.7 mmol/L), hypoalbuminemia (23.8 g/L, normal range: 42–56 g/L), hypocomplexemia (C3 0.29 g/L, normal range: 0.9–1.8 g/L; C4 0.05 g/L, normal range: 0.1–0.4 g/L), and higher erythrocyte sedimentation rate (ESR) (78 mm/h, normal range: 0–20 mm/h). Massive pleural effusion and ascites were detected. Tests for ANAs, anti SSA, anti SSB, anti-Ro52, rheumatoid factor (RF), anti-dsDNA, and lupus anticoagulant were positive. However, there are no abnormalities in renal function, electrolytes, bone marrow cytology, tumor markers, and infection screening indicators.

**Figure 1:**
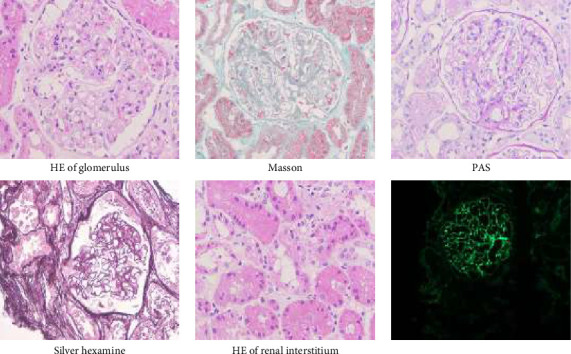
Renal pathology. HE and special staining showed that a total of 105 glomeruli were examined, among which two glomeruli were completely fibrotic, and mild mesangial proliferation in the glomerular mesangial area, 3–5 mesangial cells/proliferation area, mild segmental enlargement of the mesangial matrix, and no endothelial cell proliferation were observed. The glomerular capillary wall was slightly diffusely thickened, and the podocytes were swollen. Masson staining showed scattered osinophilic protein deposits. The renal tubular epithelial cells were swollen, granular degeneration, and protein casts and red blood cell casts were seen occasionally. There was no obvious edema, fibrosis, or a small amount of lymphocytes and mononuclear cells infiltration in the renal interstitium, and the interstitium showed no thickening of the vascular wall.

Chisholm rating of labial gland biopsy showed focal lymphocytic sialadentitis (FLS) (two stoves/4 mm^2^). Renal pathology and electron microscopy descripted lupus nephritis (Type V). Immunohistochemistry showed PLA2R (−), IgG4 (−), HBsAg (−), HBcAg (−), AA (−), kappa (+), lambda (+), CD68 (tissue cell+), FN (−), DNAJB (−), THSD7A (−), and C4d (glomerular 3+). Congo red staining was negative (Figure 1). However, a heterozygous mutation NM_0248764:c.1468C > T; (p.Arg490Cys) was identified in the *COQ8B* gene which may be associated with the clinical manifestations. The other significant variant in this gene was not identified. Finally, the diagnosis made was lupus nephritis, retinal arteriosclerosis (dual), and SLE-SS.

### 2.4. The First Treatment

Eighteen points of SLEDAI score, including eight points of visual impairment, four points of proteinuria, two points of hypocomplexemia, two points of elevated anti-dsDNA, and two points of pleural effusion, indicated severe activity of SLE. Hormone pulse combined with immunosuppressive therapy was used for the treatment. So, intravenous methylprednisolone pulse (500 mg, 3d), oral mycophenolate mofetil and hydroxychloroquine, closed thoracic drainage, and symptomatic treatment with enalapril, piperazine ferulate, albumin, and furosemide were performed. The treatment results showed negative proteinuria and disappearance of hydrothorax and ascites, but there remained a slightly elevated ESR and hypocomplementemia. Corticosteroids had been changed to oral prednisone (20 mg, tid), while other medication treatments remained unchanged.

### 2.5. Disease Changes

On the fourth day of oral therapy, the child developed abnormal mental and behavioral symptoms, including intellectual decline, delusion, impulsivity, delirium, communication difficulties, and calculation disturbance. The cerebrospinal fluid (CSF) and serum tissue–based study (TBA) tests showed moderate positive intensity. Serum antiribosomal P protein (r-RNP) and anti-GQ1b antibody IgG were positive. However, no significant abnormalities were detected in the brain magnetic resonance imaging (MRI) scanning and enhanced scan imaging. There were no abnormalities in routine, biochemical, cytological, oligoclonal zone, and culture of CSF. So, neuropsychiatric SLE (NPSLE) was considered as an additional diagnosis.

### 2.6. The Second Treatment

Based on the basis of the previous treatment, cyclophosphamide for immunosuppression, intravenous immune globulin (IVIG) to neutralize pathogenic antibodies, and olanzapine to control psychiatric symptoms were added. The child's neurological symptoms had fully recovered, lupus was under control, and all abnormal indexes were restored. She is still undergoing long-term treatment (prednisone, mycophenolate mofetil, and hydroxychloroquine) and follow-up.

## 3. Discussion

### 3.1. Diagnostic Basis

Overlap syndrome refers to a condition where the same patient experiences two or more connective tissue diseases concurrently or successively. It predominantly affects middle-aged and older individuals, with the prevalence rising as age increases. In contrast, it is relatively uncommon in children, who, due to their varied clinical presentations, are susceptible to misdiagnosis and underdiagnosis. Studies had demonstrated that the overlap of connective tissue diseases typically arises among the six traditional types, such as SLE, scleroderma, dermatomyositis, polymyositis, rheumatoid arthritis, and nodular polyarteritis. However, the current understanding of overlap syndromes has broadened beyond connective tissue diseases to encompass related conditions such as Behçet's disease and SS. Furthermore, overlaps have also been observed with other autoimmune disorders, such as Hashimoto's thyroiditis and hemolytic anemia. Clinically, the coexistence of two connective tissue diseases is the most frequent manifestation [5, 6]. It is important to note that the patients with overlap syndrome often meet the diagnostic criteria for two or more diseases and cannot be fully explained by a unifying theory. In this case, the patient's SLE manifested as proteinuria, with positive results for ANAs, anti-dsDNA, and lupus anticoagulants, accompanied by hypocomplementemia. SS, on the other hand, exhibited symptoms such as dry mouth, focal lymphocyte sialadentitis in the labial gland, and positive anti-SSA antibodies. Comparing EULAR/ACR classification criteria in SLE and SS [7], the diagnosis of SLE and SS in this patient was confirmed. But in this case of SLE-SS, the patient presented with edema and dry mouth as the primary symptoms, while dry eye symptoms were not prominent, potentially leading clinicians to overlook the diagnosis of SS.

SS is divided into primary SS (pSS) and secondary SS (sSS), where pSS does not involve additional connective tissue diseases, while sSS is often associated with other rheumatic autoimmune diseases. SLE is one of the most common autoimmune diseases associated with SS, showing certain similarities in clinical manifestations, serological characteristics, and complications. SS is diagnosed with either objective evidence of ocular and oral dryness or objective evidence of glandular parenchymal damage with serologic and histopathologic evidence of autoimmunity [8]. The serum of SS patients contains a multitude of autoantibodies, including ANAs, RF, anti-SSA, and anti-SSB, among others. Prior research had indicated that, in comparison to patients with SLE alone, the serological examination of SLE-SS is more inclined to pSS, with higher positivity rates for anti-SSA and anti-SSB antibodies, as well as elevated levels of RF and IgG [9, 10]. Consequently, autoantibodies serve as a useful tool in identifying SLE patients who may have SS as a comorbidity.

### 3.2. Renal Involvement

In comparison to the adult population, childhood-onset SLE (cSLE) tends to be more aggressive given the higher preponderance of renal and neuropsychiatric disease and increased disease activity [11]. Renal damage secondary to SLE is mainly confined to renal glomerular epithelial cells. Its main manifestation is proteinuria, and the common pathological types are Type IV and Type V. The renal damage secondary to SS is most often interstitial nephritis [12]. When the two overlap, it is possible to have concurrent manifestations of glomerular and tubular damage, with clinical manifestations mainly including renal tubularosis, glomerulonephritis, nephrotic syndrome (NS), and some with renal failure [13–15]. Therefore, when clinical suspicion of SLE-SS is present, in patients with renal involvement as the first manifestation, early renal biopsy should be performed for accurate diagnosis and effective treatment, which is paramount to halt the deterioration of renal function. In terms of treatment, there are obvious differences between lupus nephritis and renal damage secondary to SS, and the prognosis of lupus nephritis is worse than that of renal damage secondary to SS, so it is particularly important to accurately identify the primary disease according to clinical characteristics and renal biopsy. SLE-SS typically advocates for the management of lupus nephritis, utilizing adrenal cortical hormones and immunosuppressants. A prospective controlled study conducted by Karassa et al. revealed that the combination therapy of mycophenolate mofetil and glucocorticoids could effectively control the clinical manifestations of lupus nephritis [16]. The therapeutic efficacy of this combination was comparable to that of cyclophosphamide in conjunction with glucocorticoids, while the side effects associated with mycophenolate mofetil were relatively mild. For renal impairment secondary to SS, traditional Chinese medicines such as hydroxychloroquine and total paeony glycosides are commonly prescribed to regulate immune responses. In this instance of SLE-SS, the regimen combining methylprednisolone pulse therapy with mycophenolate mofetil and hydroxychloroquine was employed, yielding satisfactory outcomes in the management of renal impairment. However, based on the course of the disease may be recurrent and active, and long-term standardized follow-up treatment is essential to improve the prognosis.

### 3.3. Neuropsychiatric Manifestations

As one of the severe complications of the disease, involvement of the central or peripheral nervous system in SLE patients is termed NPSLE, for which specific diagnostic methods lack. The psychiatric symptoms in NPSLE are characterized by acute or subacute onset, lacking specificity in their manifestations. Common schizophrenic-like symptoms include behavioral disorders, hallucinations, and delusions, which may change, fluctuate, and intermittently appear throughout the course of the disease, paralleling the physical manifestations and improving alongside them. The most commonly prescribed treatment regimen is glucocorticoids combined with cyclophosphamide, with rituximab considered for some refractory cases [17, 18]. It is crucial to differentiate these symptoms from those induced by glucocorticoids, which present as mild symptoms such as euphoria, a happy mood, excessive talking, or insomnia, or severe symptoms such as inattention, fragmented hallucinations, and delusions. Psychiatric symptoms induced by glucocorticoids are highly volatile and short-lived, often resolving promptly after reducing or discontinuing glucocorticoid therapy. In this case, the patient exhibited psychiatric symptoms during the incomplete remission phase of the disease and was treated with cyclophosphamide pulse, IVIG, and antipsychotic therapy, without reduction or discontinuation of glucocorticoids. After recovery, no residual psychiatric symptoms were observed, confirming the NPSLE diagnosis. Given the high disability and mortality rates associated with central nervous system involvement in SLE patients, close follow-up is necessary. The absence of specific follow-up duration and long-term outcome is a limitation of this patient.

## 4. Conclusion

In summary, the following points are our experiences: (1) The clinical manifestations of overlap syndrome are complex and varied, mainly depending on the coexisting disease. So it is not possible to meet one diagnosis in patients with rheumatic diseases, which require necessary laboratory examinations to avoid missing the diagnosis of another coexisting rheumatic disease. (2) It is relatively common for SLE-SS to develop secondary renal damage clinically, but it is difficult to distinguish the primary disease, and the prognosis varies greatly. Therefore, for such patients, early renal biopsy to clarify the primary disease has become the key to treatment. (3) For patients with mental abnormalities or symptoms of neurological dysfunction, it is easy to be confused with intracranial infectious diseases and drug factors resulting in misdiagnosis and mistreatment. Therefore, it is imperative for clinicians to give them utmost attention, thoroughly gather medical histories, perform detailed physical examinations, conduct necessary laboratory tests, enhance the precision of early clinical diagnosis and treatment, and adopt appropriate therapeutic strategies. These measures are crucial for improving patient outcomes.

## Data Availability

Data sharing is not applicable to this article as no new data were created or analyzed in this study.

## Nomenclature

SLE: Systemic lupus erythematosus
SS: Sjögren's syndrome
RF: Rheumatoid factor
SRNS: Steroid-resistant nephrotic syndrome
FSGS: Focal segmental glomerulosclerosis
